# Quantification of Oxygenation and Oxygen Consumption Rates in the Mouse Brain Based on Tissue Oxygen Level‐Dependent (TOLD) MRI

**DOI:** 10.1002/nbm.70104

**Published:** 2025-07-22

**Authors:** Raj Kumar Parajuli, Hiromi Sano, Megumi Ueno, Shan Gao, Saaya Suzuki, Akira Sumiyoshi, Kensuke Osada, Takayuki Obata, Ichio Aoki, Ken‐ichiro Matsumoto

**Affiliations:** ^1^ Institute for Quantum Medical Science, National Institutes for Quantum Science and Technology (QST) Chiba Japan; ^2^ Sydney Imaging Core Research Facility The University of Sydney Sydney New South Wales Australia; ^3^ Department of Molecular Imaging and Theranostics, Applied MRI Research Group Institute for Quantum Medical Science, QST Chiba Japan; ^4^ Department of Radiation Regulatory Science Research, Quantitative RedOx Sensing Group Institute of Radiological Science, QST Chiba Japan; ^5^ Department of Molecular Imaging and Theranostics, Functional and Molecular Imaging Group Institute for Quantum Medical Science, QST Chiba Japan; ^6^ Department of Accelerator and Medical Physics, Radiation Effect Research Group Institute for Quantum Medical Science, QST Chiba Japan; ^7^ Department of Molecular Imaging and Theranostics, Diagnostic and Therapeutic Nuclear Medicine Group Institute for Quantum Medical Science, QST Chiba Japan

**Keywords:** brain, oxygen consumption rate, oxygen metabolism, oxygenation rate, T_1_‐weighted contrast, tissue oxygen level‐dependent signal

## Abstract

A noninvasive method to estimate oxygenation and oxygen (O_2_) consumption rates in mouse brain tissue based on T_1_‐weighted tissue oxygen level‐dependent (TOLD) MRI was developed. The aim of the present study was to estimate the kinetics of O_2_ in tissue in order to facilitate the planning of radiation therapy. The relationship between the proton T_1_ relaxation rate and O_2_ concentrations was examined in water and corn oil samples, which are models of the aqueous and lipidic phases of cell components. T_1_ maps of the mouse brain were obtained under breathing high oxygen concentration gas, carbogen (95% O_2_ + 5% CO_2_), or 100% O_2_, and shifts in the concentration of O_2_ from the physiological state in the brain were then estimated. Pseudo‐TOLD signals in blood samples, hemoglobin, and myoglobin aqueous solutions under oxygenation were measured to examine the effects of heme iron on T_1_. O_2_ metabolism in mouse brains was evaluated by the enhancement/diminishment rates of the TOLD signal when the breathing gas was switched from air to carbogen or 100% O_2_ and when it was switched back to air. Carbogen or 100% O_2_ inhalation increased cortical O_2_ levels by 0.28 or 0.26 mmol/L, respectively. By analyzing the time course profile of the TOLD signal, the oxygenation rate, oxygenation level, and O_2_ consumption rate were successfully estimated. TOLD MRI has potential as a useful tool to estimate the quantitative indices of oxygenation and O_2_ consumption rates in brain tissue.

## Graphical abstract

The oxygenation rate, *k*_ox_, and the O_2_ consumption rate, *k*_oc_, in the mouse brain parenchyma were estimated based on the increasing and decreasing phases of the tissue oxygen level dependent (TOLD) signal, which is oxygen‐induced enhancement of the T_1_‐weighted signal in the tissue.

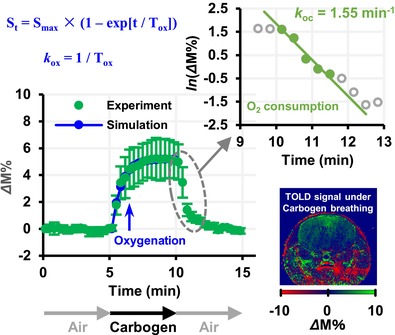

**Abbreviations**
BOLD: blood oxygen level‐dependent
deoxy‐Hb: deoxyhemoglobin
EPR: electron paramagnetic resonance
LiPc: lithium phthalocyanine
oxy‐Hb: oxyhemoglobin
PET: positron emission tomography
PTFE: polytetrafluoroethylene
pO_2_
: partial pressure of oxygen
R_1_
: T_1_ relaxation rate
r_1_
: T_1_ relaxivity
RBC: red blood cell
RF: radio frequency
TOLD: tissue oxygen level‐dependent

## 1 Introduction

An insufficient oxygen (O_2_) supply due to an immature vascular structure in tumor tissue induces tissue hypoxia [1, 2]. Hypoxia shifts energy production in the cell from the TCA cycle to glycolysis [3]. Glycolysis results in lactate accumulation and causes a low pH environment [4]. Hypoxia may lead to treatment resistance and modify the redox status of tumor tissue [5]. Therefore, the quantitative detection of the partial pressure of oxygen (pO_2_) and evaluations of hypoxia in tumor tissues are important for planning cancer radiotherapy [6, 7, 8, 9, 10, 11, 12, 13, 14]. Accurate and high‐resolution mapping techniques for hypoxia, pO_2_, and/or O_2_ kinetics in tumor tissues may facilitate the success of treatment.

Positron emission tomography (PET) with molecular probes has been developed to detect tumor hypoxia [6, 8, 14]. However, the ability of PET techniques to map hypoxic regions in tumors based on the specific chemical binding reaction of the probe under low O_2_ conditions is limited, and background signal contamination is unavoidable due to the pharmacokinetics of the contrast agent. Although quantitative O_2_‐mapping methods based on electron paramagnetic resonance (EPR) have been developed [7, 8, 9, 10] and used on small animals, difficulties are associated with their application to humans due to weak signal intensity as a result of a low radio frequency (RF) with a larger resonator [15].

In contrast, MRI has high spatial resolution and provides three‐dimensional images of deep regions in humans. Blood O_2_ level‐dependent (BOLD) MRI has been utilized to investigate tissue O_2_ requirements, particularly in brain functions. The BOLD signal is the response of a T_2_*‐weighted signal induced by a population shift from paramagnetic deoxyhemoglobin (deoxy‐Hb) to diamagnetic oxyhemoglobin (oxy‐Hb), which decreases magnetic susceptibility and extends T_2_* [16, 17]. Although O_2_ kinetic parameters have been analyzed using T_2_*‐based BOLD MRI [18, 19], BOLD signal intensity is less quantitative for pO_2_ because it depends not only on tissue pO_2_ but also on blood volume. On the other hand, O_2_ is a stable free radical species with two unpaired electrons that enhance T_1_‐weighted MRI contrast [19, 20, 21, 22, 23] and is less dependent on blood volume. Molecular O_2_ may enhance the spin–lattice relaxation rate similar to other paramagnetic contrast agents, such as gadolinium compounds [24]. Therefore, T_1_‐based tissue O_2_ level‐dependent (TOLD) MRI detects direct responses in tissue pO_2_ and is an important quantitative parameter [20].

Tissue T_1_ shortening and T_1_‐weighted signal enhancement induced by breathing hyperoxic gas have been demonstrated in both animals and human volunteers. Beeman et al. [25] reported a linear relationship between R_1_ values and pO_2_ in the brains of mice breathing gas containing a specific concentration of O_2_. Winters et al. [26] showed T_1_ shortening in normal abdominal tissues, including the liver, kidney, and paraspinal muscle, of rabbits breathing 100% O_2_, whereas T_1_ extension was observed under breathing 10% CO_2_ in air as well as carbogen (10% CO_2_ + 90% O_2_). O'Connor et al. [27] examined the abdominal organs of healthy human volunteers at 1.5 T and found a positive shift in the T_1_‐weighted signal in the spleen, liver, paraspinal muscle, and renal cortex under breathing 100% O_2_. The switching of gas from 100% O_2_ to carbogen resulted in an additional signal increase in the liver, but not in the paraspinal muscle or renal cortex, and a slight decrease in the spleen [27]. Tadamura et al. [28] also detected T_1_‐weighted signal enhancement in the spleen and myocardium under breathing 100% O_2_ in healthy human volunteers. Furthermore, Jones et al. [29] reported positive T_1_‐weighted signal enhancement in the renal cortex of human volunteers under breathing 100% O_2_.

The use of TOLD in the planning of radiation therapy and controlling tumor/cancer is already several years in development. Salem et al. [30] proposed that the oxygen refractory volume of lung tumor tissue under a 100% O_2_ challenge in animals and/or human patients measured by mapping R_1_ changes from that under breathing air has potential as a marker of hypoxic volume. McCabe et al. [31] similarly estimated hypoxic fractions in the oropharyngeal tumors of patients by mapping the insensitive tissue volume of T_1_ shortening caused by an O_2_ challenge. Dubec et al. [32] also evaluated tumor hypoxia in patients with oropharyngeal tumors and found that radiation therapy modified hypoxic volume. Arai et al. employed oxygen‐sensitive MRI to differentiate radiation‐responsive from nonresponsive tumor areas based on tumor hypoxia and to predict tumor control.

Squamous cell carcinoma tumor tissue planted in the mouse hind leg showed a positive TOLD signal under high concentration O_2_ breathing [20]. The amplitude of the positive TOLD signal, that is, the enhancement of the T_1_‐weighted signal, is associated with an increase in the concentration of O_2_ in the target tissue. However, in normal healthy muscle tissue, a negative shift in the TOLD signal, that is, the attenuation of the T_1_‐weighted signal, under high concentration O_2_ breathing has been observed. A negative TOLD signal makes the evaluation of pO_2_ variations in muscle tissue challenging. The incidence of the negative TOLD signal may be dependent on the tissue/organ. Although the T_1_ value observed in normal muscle tissue was stable or slightly extended under high concentration O_2_ breathing [20, 34], it remains unclear whether the negative TOLD signal in normal muscle tissue reflects variations in the concentration of O_2_ in the tissue. A population shift from paramagnetic to diamagnetic heme iron may also extend the T_1_ value in muscle under O_2_ breathing because muscle is rich in myoglobin (Mb). Therefore, an O_2_‐induced positive TOLD signal may be observed in tissue containing low heme iron, such as the brain.

Effective oxygenation procedures that cancel hypoxia and enhance the effects of radiotherapy and/or chemotherapy in tumor tissues have been investigated. Breathing carbogen or 100% O_2_ represents an effective approach to increase tissue oxygenation [20, 24, 25, 26, 27, 28, 29, 30, 31, 32, 33, 34]. The T_1_‐based oxygenation responses of some tissues/organs do not always depend on the O_2_ concentration in the breathing gas [20, 26, 34]. To select better oxygenation treatments for the brain, the oxygenation ability of carbogen and 100% O_2_ was compared.

In the present study, a method was developed to evaluate O_2_ metabolism, that is, oxygenation and O_2_ consumption, in brain tissue. Linearity between dissolved O_2_ concentrations and the O_2_‐induced positive TOLD signal in the sample was confirmed. The mechanisms underlying the negative shift in the TOLD MRI signal in muscle under O_2_ breathing were then elucidated. To establish whether the TOLD signal observed in brain tissue was positive and dependent on O_2_, the relationship between the shift in the T_1_‐weighted signal and changes in the T_1_ value in the mouse brain induced by switching the breathing gas from air to high concentration O_2_ was investigated. A kinetic analysis of the increasing/decreasing phase of the TOLD signal induced by switching the breathing gas of mice was performed.

## 2 Theory

### 2.1 Estimation of Changes in pO_2_ in the Brain Based on the Aqueous/Lipidic Volume Ratio

To assess pO_2_ shifts in the brain, the volume ratio of water and lipid components in tissue was estimated in vitro. A shift in the T_1_ relaxation rate (R_1_), that is, *Δ*R_1_, in a sample induced by increasing/decreasing pO_2_ is converted to a shift in pO_2_ based on the T_1_ relaxivity (r_1_) of O_2_. In in vivo experiments, the tissue T_1_ relaxation time (T_1EXP_) in a pixel/voxel comprises several different relaxation components (Equation 1).

(1) $$1/T_{\text{1EXP}}=1/T_{\text{1A}}+1/T_{\text{1B}}+1/T_{\text{1C}}+\ldots .$$

In mammalian cells, 70%–80% of cell components are water, while 30%–50% of residual cell components other than water are lipids. Therefore, the T_1_ value in tissue (T_1T_) is assumed to mainly comprise aqueous (T_1A_) and lipidic (T_1L_) components. By using T_1_ values in water and corn oil as T_1A_ and T_1L_, respectively, the ratio of aqueous and lipidic components in the mouse brain is roughly predicted (Equations 2 and 3).

(2) $$1/T_{\text{1T}}=1/T_{\text{1A}}\times V_{A}+1/T_{\text{1L}}\times V_{L}$$

(3) $$V_{A}+V_{L}=1$$

where V_A_ and V_L_ are the ratios of aqueous and lipidic volumes, respectively.

When r_1_ of O_2_ in water (r_1A_) and corn oil (r_1L_) is used as the estimation of r_1_ in the aqueous and lipidic phases in tissue, a shift in pO_2_ (*Δ*O_2_) in tissue may be expected as Equation (4) based on experimentally observed tissue *Δ*R_1_.

(4) $$\Delta O_{2}=\text{tissue}\;\Delta R_{1}/r_{\text{1A}}\times V_{A}+\text{tissue}\;\Delta R_{1}/r_{\text{1L}}\times V_{L}$$

### 2.2 Estimation of Tissue Oxygenation and O_2_ Consumption Rates

Brain tissue oxygenation and O_2_ consumption rates may be estimated by the time course of the tissue T_1_‐weighted signal enhancement induced by O_2_, that is, the TOLD signal, with the assumption that the TOLD signal in brain tissue is linearly related to pO_2_. In the present study, the oxygenation rate, *k*_ox_, the maximum level of the TOLD signal, S_max_, and the O_2_ consumption rate, *k*_oc_, were proposed as indices for O_2_ metabolism. An exponential function (Equation 5) is fitted on the increasing phase of the TOLD signal, and the parameters T_ox_ and S_max_ are estimated.

(5) $$S_{t}=S_{\max}\times \left(1—\exp.\left[t/T_{\mathrm{ox}}\right]\right)$$

where S_max_ is the maximum level of the T_1_‐weighted signal, and T_ox_ is the time required to increase the TOLD signal to 63.2% of S_max_. The reciprocal of T_ox_, that is, *k*_ox_ = 1/T_ox_, estimates the increment rate of the TOLD signal, that is, the oxygenation rate. *k*_oc_ may be estimated from the slope of the semi‐logarithmic plot of the decreasing phase of the TOLD signal.

## 3 Materials and Methods

### 3.1 Chemicals

Carbogen gas (a mixture of 95% O_2_ and 5% CO_2_) was purchased from Tomoe Shokai Co. Ltd. (Tokyo, Japan). Hemoglobin (Hb) and Mb were purchased from Sigma‐Aldrich Co. (St. Louis, MO). Lithium phthalocyanine (LiPc) crystals were synthesized according to a previously reported method [35]. Other chemicals were of analytical grade.

### 3.2 Animals

Seven‐week‐old female C3H/HeSlc mice were supplied by Japan SLC Inc. (Shizuoka, Japan). Animals were housed five per aluminum cage with a woodchip bed in climate‐controlled (23 ± 1°C and 55 ± 5% humidity) circadian rhythm‐adjusted (12‐h light–dark cycle) rooms and were allowed food and water *ad libitum*. Mice were used in experiments after habituation for 1 week. The total number of mice used was 12, with a mean body weight of 19.9 ± 0.58 g. Experiments (No. 22‐1010) were performed in compliance with the Guidelines for Animal Care and Use at the National Institute of Radiological Sciences and the National Institutes for Quantum Science and Technology (NIRS/QST) and were approved by the Animal Use Committee of the NIRS/QST. Detailed information on animal handling and experiments was provided according to the ARRIVE guidelines [36].

### 3.3 Blood Samples

Eight‐week‐old mice were anesthetized by 1.5%–2.0% isoflurane using air as the carrier gas. Blood was collected from the abdominal artery 1 day before the experiment. A portion of blood was centrifuged at 1000× *g* for 10 min to obtain plasma and red blood cell (RBC) fractions. Whole blood and fractions were kept on ice until MRI experiments.

### 3.4 O_2_ Solubility in Corn oil

O_2_ solubility in corn oil used in the present study was assessed by EPR oximetry. LiPc is a crystalized solid paramagnetic species, the EPR linewidth of which is broadened by a spin–spin interaction with O_2_ [35, 37]. LiPc crystals were placed into a 24‐cm piece of gas‐permeable polytetrafluoroethylene (PTFE) tubing (i.d.: 0.81 ± 0.025 mm, wall thickness: 0.051 ± 0.013 mm). PTFE tubing was purchased from ZEUS (Orangeburg, SC). An ~80‐μL aliquot of milli‐Q water or corn oil was carefully drawn into PTFE tubing, and LiPc crystals were immersed in the liquid inside. The PTFE tubing was placed in a special quartz sample holder (Figure S1). The gas line was connected to the bottom of the sample holder, which had a hole for gas flow. The sample holder was set in the X‐band (9.4 GHz) EPR TE‐mode cavity, and the position of LiPc crystals was adjusted to the center of the sensitive region of the cavity. The EPR cavity including the sample was warmed to ~30°C using an IR lamp and hot air flow. The EPR linewidth of LiPc was measured under N_2_, air, or 100% O_2_ gas flow (1 L/min).

EPR data acquisition was performed using an X‐band EPR spectrometer (JEOL, Tokyo, Japan), which was controlled by the WIN‐RAD ESR Data Analyzer System (Radical Research Inc., Hino, Tokyo). The singlet EPR spectrum of LiPc was scanned under the following conditions: microwave frequency, 9.45 GHz; microwave power, 1 mW; center magnetic field, 337.3 mT; field sweep width, ±0.25 mT; field sweep resolution, 4096 points; sweep time, 1 min; time constant, 0.01 s; field modulation frequency, 100 kHz; field modulation width, 0.0063 mT. The EPR spectra acquired were analyzed using an in‐house line‐fitting program [38], and the mixed Gaussian/Lorentzian line shape was fit with the best suited mixing rate. The linewidth of the line shape was measured.

### 3.5 Measurement of T_1_ Relaxivity of O_2_ and T_1_‐Weighted Signal Enhancement by O_2_ (A Phantom Experiment)

As a simple model of T_1_ components in the aqueous and lipidic phases of a cell, the T_1_ relaxivity of O_2_ was estimated in water and corn oil. Linoleic acid‐rich corn oil was selected as a model environment of the lipidic phase. The T_1_‐weighted signal enhancements induced were dependent on O_2_ concentrations in these solvents (pseudo‐TOLD signal).

An ~80‐μL aliquot of milli‐Q water was drawn into a 24‐cm piece of PTFE tubing, which was folded at the center to hold the water inside. PTFE tubing was placed in a quartz tube (i.d. = 3.6 mm), which was connected to the gas line at one end. The quartz tube was set in the 7 T MRI scanner. The sample temperature during the scan was maintained at ~30°C using hot air flow. Figure shows a schematic of the experimental setting. A T_1_ map of a cross‐section of the phantom was measured under nitrogen (N_2_), air, carbogen, or 100% O_2_ gas flow (400 mL/min) conditions. T_1_‐weighted images of the phantom were repeatedly scanned for 21 min, during which air was flowed for the initial 5 min, and the gas was then switched to carbogen, 100% O_2_, or N_2_ for the remaining 16 min. The same experiment was repeated using corn oil, 5 mM Hb aqueous solution, or 5 mM Mb aqueous solution instead of milli‐Q water. Using mouse whole blood, blood plasma, and the RBC fraction, the pseudo‐TOLD signal induced by switching gas from N_2_ to air was observed. Instrument details and scanning parameters are described below.

### 3.6 In Vivo Experiment 1: Effects of Carbogen and 100% O_2_ on T_1_ Relaxation Rates and TOLD Signals in Mouse Brains

To confirm the quantitative estimations of oxygenation and O_2_ consumption in brain tissue, changes in T_1_ relaxation rates (R_1_ = 1/T_1_) and T_1_‐weighted signal enhancements in the brain tissue of mice breathing gas with a high O_2_ concentration as the carrier of anesthesia were measured. The settings of mice, instrument details, and scanning parameters are described below.

The left panel in Figure S3 shows a flow diagram of this experiment. A T_1_ map was generated under air breathing. T_1_‐weighted images of the corresponding mouse head region were repeatedly scanned, during which the mouse inhaled air for the initial 5 min and carbogen for the next 16 min. A T_1_ map was then generated under carbogen breathing. The breathing gas was subsequently switched back to air, and after a 5‐min interval, a T_1_ map was obtained under air breathing. Repeated T_1_‐weighted scanning was started, during which mice breathed air for 5 min and then 100% O_2_ for the next 16 min. A T_1_ map was obtained under 100% O_2_ breathing. The settings of mice, instrument details, and scanning parameters are described below.

### 3.7 In Vivo Experiment 2: Estimation of O_2_ Metabolism in the Mouse Brain

To examine oxygenation and O_2_ consumption in the mouse brain, carbogen‐induced TOLD scanning was performed on mice. A mouse was set on the MRI scanner, and repeated T_1_‐weighted scanning was initiated under air breathing. The mouse breathed air for the initial 5 min followed by carbogen for the next 5 min. The carrier gas was then switched back to air for the last 5 min. The settings of mice, instrument details, and scanning parameters are described below. An experimental flow diagram is shown in the right panel of Figure S3.

### 3.8 Mouse Preparation and the MRI Instrument

A mouse was anesthetized by 1.5%–2.0% isoflurane using air as the carrier gas. The mouse was placed in the prone position on a custom‐built body cradle and immobilized using adhesive tape. The respiration rate was monitored using a respiration sensor (MP150, Biopac System Inc., Goleta, CA, USA) and regulated at 60–80 breaths per minute. Body temperature was monitored with a rectal probe (FOT‐L‐SD‐CI‐FI‐MI‐R2‐ST, FISO Technologies Inc., Germany) and regulated at 36.0 ± 2.0°C using a water‐circulating pad and warm air circulation system. MRI data were acquired using a horizontal 7.0‐T MRI system (BioSpec 70/20 USR, Bruker Biospin, Ettlingen, Germany) with a cryogenic RF coil (cryo‐probe, Bruker Biospin) for transmission and reception in MRI. All MRI data were acquired and reconstructed using ParaVision software (Ver. 7, Bruker Biospin). Following standard adjustment routines, scout scans were used for accurate positioning inside the magnet.

### 3.9 T_1_ and R_1_ Mapping

T_1_ mapping of the brain was performed using Rapid Acquisition with Refocused Echoes and variable TR sequencing with 12 steps of TR (15,000, 8000, 5500, 4000, 3000, 2000, 1600, 1200, 800, 500, 200, and 100 ms). TE was 40 ms, FA was 180°, and slice thickness was 1 mm. MRI images were reconstructed using ParaVision‐7 software, exported in the DICOM format, and converted to the NIfTI format using dcm2niix software (https://github.com/rordenlab/dcm2niix). T_1_ maps were generated using the MRI Analysis Calculator plugin (produced by Karl Schmidt in HypX Laboratory, Brigham & Women's Hospital, Boston, MA) worked on the ImageJ software package (http://rsb.info.nih.gov/ij/). T_1_ maps were converted to corresponding R_1_ maps, that is, maps of the reciprocal values of T_1_.

### 3.10 TOLD Imaging

In the phantom experiment and in vivo experiment 1, T_1_‐weighted images of the corresponding region were repeatedly scanned by multiple‐slice fast low angle shot (FLASH) sequencing (TR = 164.0 ms, TE = 2.3 ms, FA = 45°, Nex = 2) under anesthesia with 1.5%–2% isoflurane. The number of slices was 7. Slice thickness was 1 mm and the slice interval was 1 mm. The scan time for each frame was 43 s, and a dataset comprising 29 frames was obtained.

In in vivo experiment 2, TOLD imaging was performed using the FLASH sequence. The parameters for FLASH scanning in experiment 2 were as follows: TR = 125.0 ms, TE = 1.6 ms, FA = 45°, Nex = 2, number of slices = 7, slice thickness of 1 mm, and a slice interval of 1 mm. The scan time for each frame was 20 s and a dataset comprising 45 frames was obtained.

A set of T_1_‐weighted MRI images was reconstructed using ParaVision‐7 software, exported in the DICOM format, and converted to the NIfTI format using dcm2niix software (https://github.com/rordenlab/dcm2niix). Motion corrections were performed using 3dvolreg (AFNI, https://afni.nimh.nih.gov/), an automated alignment program that co‐registered each volume in the time series to the first volume. Time‐course images were analyzed using ImageJ. The pixel‐wise percentage enhancement of the T_1_‐weighted signal, *Δ*M%_x,y_, was calculated as follows:
(6)

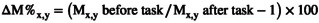

where M_x,y_ is the image intensity of the pixel in the slice at the (x, y) coordinate. The shift in *Δ*M% induced by the oxygenation treatment was called the TOLD signal.

### 3.11 Statistical Analysis

In comparisons between two factors, for example, when two values before and after a task were compared, the significance of differences was estimated by the *t*‐test using the TTEST function in Microsoft Excel 2010. The “tail” was 1 (one‐tailed distribution) when increasing or decreasing values caused by the task were predictable, and T_1_ changes by the O_2_ task were compared. The “tail” was 2 (two‐tailed distribution) when the difference in values caused by the task was expected. The “type” was 2 (equal variance) or 3 (unequal variance), which was estimated using the FTEST function. The “type” was 1 for paired datasets when changes in an identical image were compared. Significance estimated by the *t*‐test was accepted when the *p* value was < 0.05.

The significance of differences in comparisons among multiple factors, for example, when differences among the effects of N_2_, air, carbogen, and 100% O_2_ were compared, was estimated by the Tukey–Kramer test with α = 0.05. The Tukey–Kramer test was conducted using a self‐built Excel spreadsheet. Tukey–Kramer tables were downloaded from a website (https://imnstir.blogspot.com/2012/03/exceltukey.html).

## 4 Results

### 4.1 Estimation of r_1_ Values of O_2_ in Water and Corn Oil

To calculate the shift in tissue pO_2_, *Δ*O_2_, the r_1_ values of O_2_ in water and corn oil were observed as estimates of the r_1_ values of the aqueous and lipidic phases in tissue.

O_2_ solubility in water and corn oil was initially estimated. The EPR linewidth of LiPc in corn oil under N_2_ gas flow was 0.0092 ± 0.0002 mT, which was similar to that in water under N_2_ gas flow (0.0094 ± 0.0002 mT). Corn oil did not physically affect the EPR linewidth of LiPc. Figure S4A,B shows the relationships between EPR linewidths and pO_2_ in water and corn oil, respectively. Because both plots were similar, O_2_ solubility in water and corn oil was equivalent.

**FIGURE 1:**
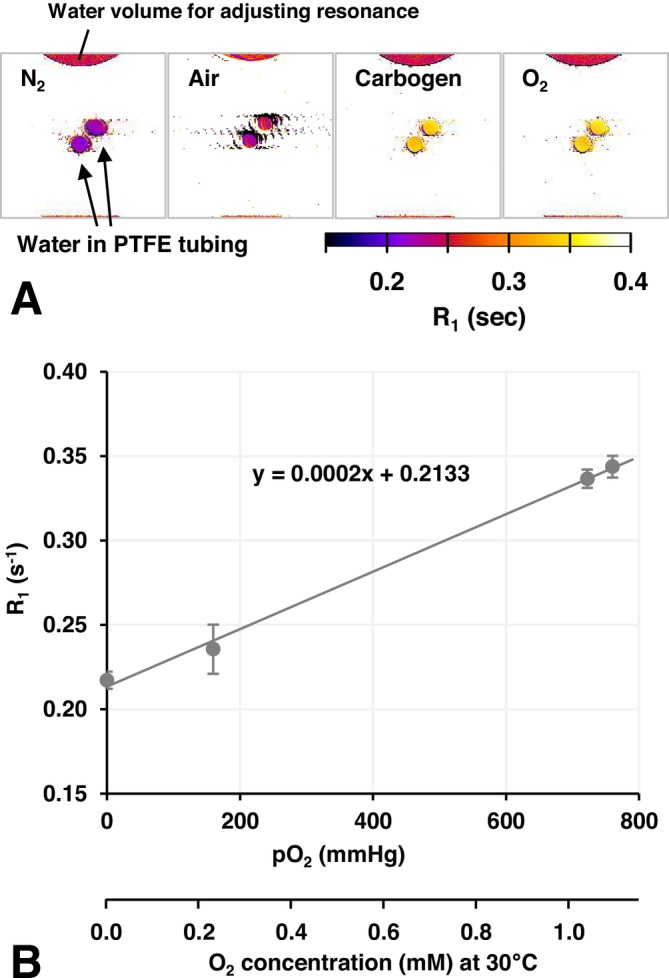
Estimation of T_1_ relaxivity, the r_1_ value, of O_2_ in water at 7 T. (A) R_1_ maps observed under N_2_, air, carbogen, and 100% O_2_ gas flow. (B) Relationship between R_1_ of water and pO_2_ of gas dissolved in water. O_2_ concentrations at 30°C were calculated with the assumption that the water sample was saturated with the gas. Values are indicated as the average ± SD. The r_1_ value was estimated from the slope of the regression line.

Figure 1 shows the relationship between O_2_ concentrations and quantitative longitudinal relaxation rates (R_1_) in water samples. R_1_ maps of cross‐sections of water samples were generated under different gas conditions (Figure 1A). R_1_ values in water were obtained and plotted versus pO_2_ of the corresponding gas (Figure 1B). T_1_ and R_1_ values in water and corn oil samples under several gas conditions are shown in Table 1. The T_1_ relaxivity of O_2_ in water at 7 T was estimated as 1.71 × 10^−4^ (mmHg)^−1^ s^−1^, which corresponded to 0.118 mM^−1^ s^−1^ with the assumption that the water sample was saturated with the gas at 30°C.

**TABLE 1:** T_1_ and R_1_ values of water and corn oil at 7 T under several different gas flow conditions.

Sample		N_2_	Air	Carbogen	O_2_
Water	T_1_ (s)	4.61 ± 0.11	4.25 ± 0.26	2.97 ± 0.05	2.91 ± 0.05
	R_1_ (s^−1^)	0.22 ± 0.01	0.24 ± 0.01	0.34 ± 0.01	0.34 ± 0.01
Corn oil	T_1_ (s)	0.67 ± 0.01	0.60 ± 0.01	0.48 ± 0.01	0.47 ± 0.01
	R_1_ (s^−1^)	1.50 ± 0.02	1.66 ± 0.02	2.10 ± 0.02	2.14 ± 0.03

*Note:* Values indicate the average ± SD of three experiments.

**FIGURE 2:**
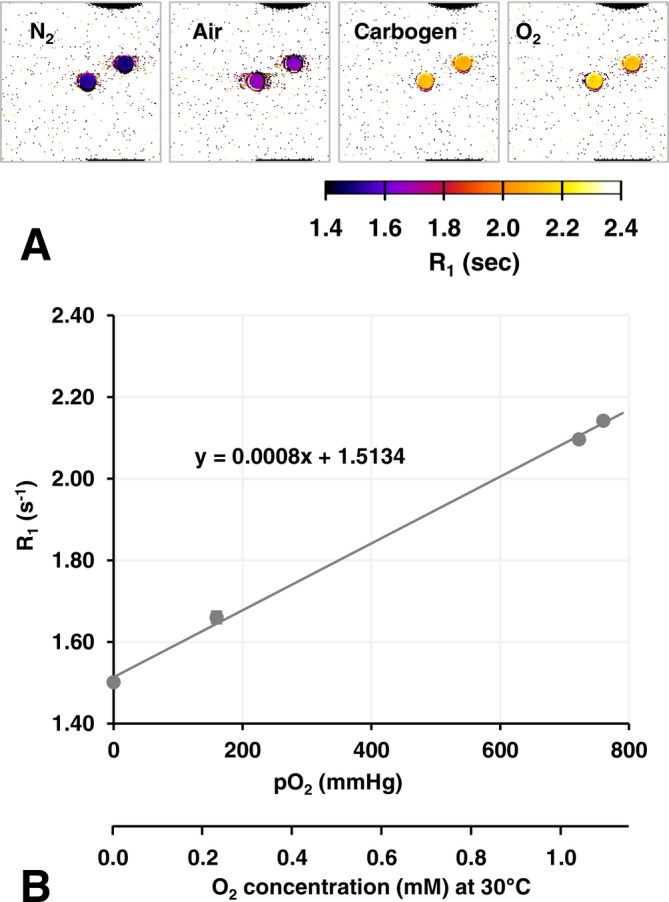
Estimation of T_1_ relaxivity, the r_1_ value, of O_2_ in corn oil at 7 T. (A) R_1_ maps observed under N_2_, air, carbogen, and 100% O_2_ gas flow. (B) Relationship between R_1_ of corn oil and pO_2_ of gas dissolved in corn oil. O_2_ concentrations in corn oil at 30°C were experimentally observed. Values are indicated as the average ± SD. The r_1_ value was estimated from the slope of the regression line.

R_1_ maps of corn oil samples were obtained under different gas conditions (Figure 2A). Figure 2B shows the relationship between the R_1_ values of corn oil and pO_2_ of the gas. The r_1_ of O_2_ in corn oil at 7 T was estimated as 8.20 × 10^−4^ (mmHg)^−1^ s^−1^, which corresponded to 0.569 mM^−1^ s^−1^ at 30°C with the assumption that O_2_ solubility was similar in corn oil and water.

### 4.2 Measurement of T_1_‐Weighted Signal Enhancement by O_2_ in Water and Corn Oil

**FIGURE 3:**
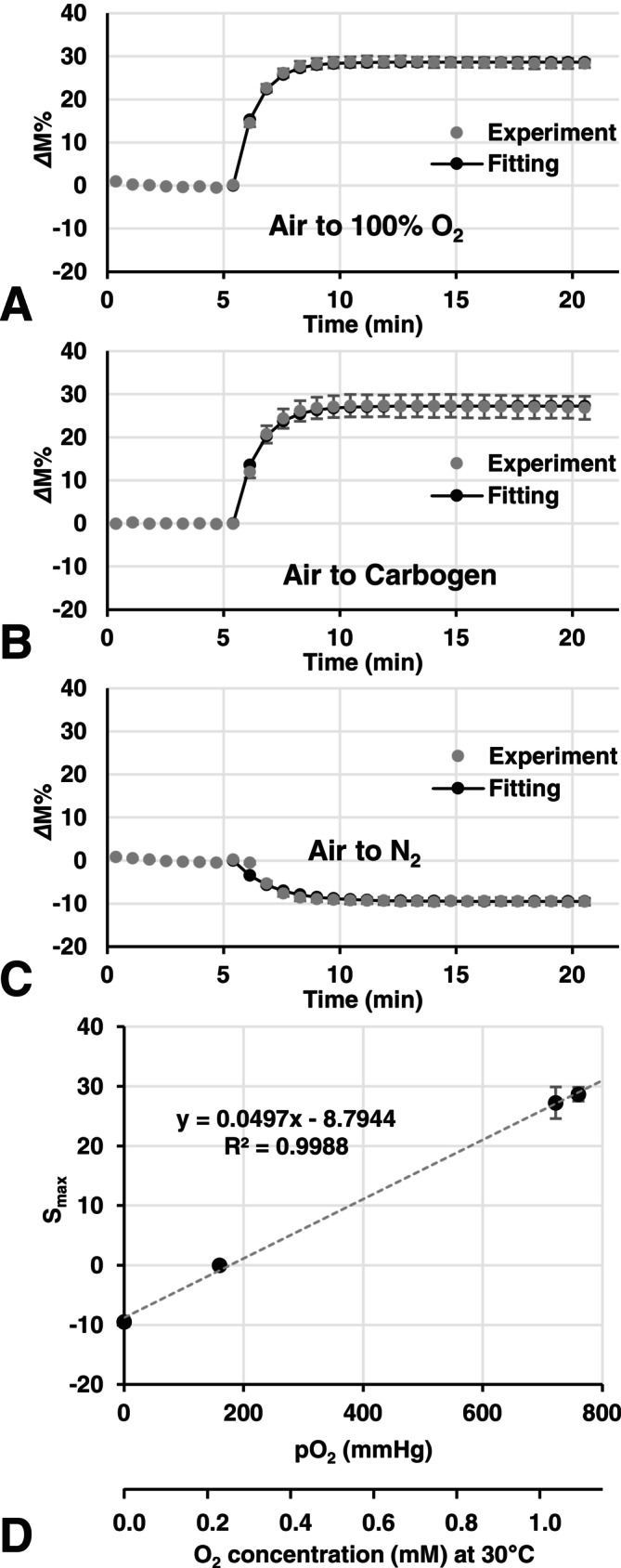
Time courses of the TOLD signal, that is, T_1_‐weighted signal enhancement/diminishment induced by pO_2_ changes, in the water sample. The *Δ*M% values observed were plotted versus time. (A) TOLD signal enhancement in the water sample induced by switching gas flow from air to 100% O_2_. (B) TOLD signal enhancement in the water sample induced by switching gas flow from air to carbogen. (C) TOLD signal diminishment in the water sample induced by switching gas flow from air to N_2_. The indices of oxygenation, that is, *k*_ox_ (= 1/T_ox_) and S_max_, were observed by fitting Equation (2) to the increasing phase of the plot. (D) Relationship between S_max_ values and pO_2_ of gases.

Figure 3A–C shows the time courses of T_1_‐weighted signal enhancement/diminishment in the phantom, that is, a pseudo‐TOLD signal, induced by a rapid change in the dissolved O_2_ concentration in water samples during repeated FLASH scanning. The pseudo‐TOLD signal rapidly increased when the gas was switched from air to carbogen or 100% O_2_ (Figure 3A,B). On the other hand, the pseudo‐TOLD signal rapidly decreased when the gas was switched from air to N_2_ (Figure 3C).

The time course of the pseudo‐TOLD signal in the phantom was manipulated by fitting Equation (4). Figure 3D shows a plot of S_max_ values versus the pO_2_ of the corresponding gases. In this plot, the S_max_ value for air was 0, that is, the baseline. The relationship between S_max_ and O_2_ concentrations was almost linear. *k*_ox_ values observed under 100% O_2_, carbogen, and N_2_ flow were 1.06 ± 0.045, 0.96 ± 0.026, and 0.63 ± 0.012 min^−1^, respectively.

**FIGURE 4:**
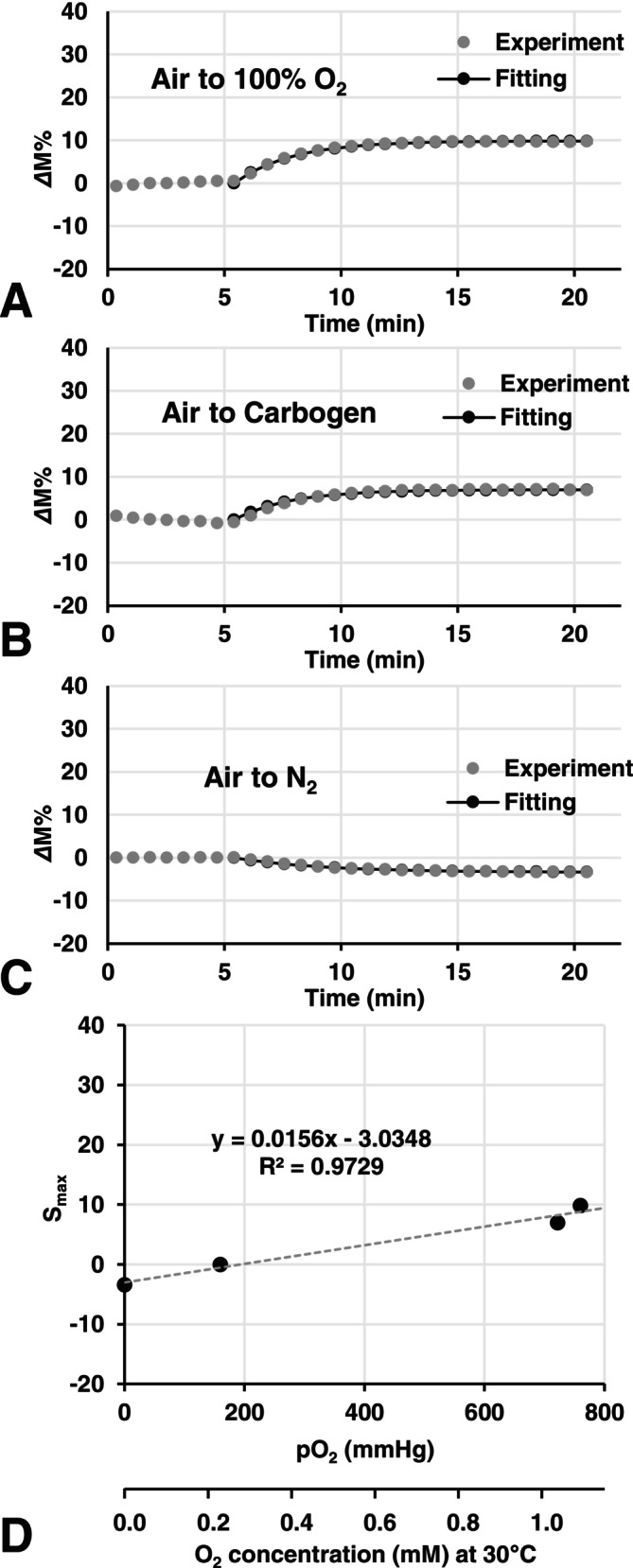
Time courses of the TOLD signal induced by pO_2_ changes in corn oil. *Δ*M% values, the enhancement ratio of the T_1_‐weighted signal, were plotted versus time. (A) TOLD signal enhancement in the corn oil sample induced by switching gas flow from air to 100% O_2_. (B) TOLD signal enhancement in the corn oil sample induced by switching gas flow from air to carbogen. (C) TOLD signal diminishment in the corn oil sample induced by switching gas flow from air to N_2_. The indices of oxygenation, that is, *k*_ox_ (= 1/T_ox_) and S_max_, were observed by fitting Equation (2) to the increasing phase of the plots. (D) Relationship between S_max_ values observed in corn oil and pO_2_ of gases.

Figure 4A–C shows the time courses of the pseudo‐TOLD signal induced by abrupt changes in pO_2_ in corn oil samples during repeated FLASH scanning. The pseudo‐TOLD signal in corn oil increased when the gas was switched from air to carbogen or 100% O_2_ (Figure 4A,B) and decreased when the gas was switched from air to N_2_ (Figure 4C). S_max_ levels were smaller than those in water samples. *k*_ox_ values were 0.41 ± 0.019, 0.41 ± 0.018, and 0.26 ± 0.029 min^−1^ for 100% O_2_, carbogen, and N_2_, respectively. Figure 4D shows a plot of S_max_ values in corn oil versus pO_2_ of the corresponding gas. The slope of Figure 4D was smaller than that of water (Figure 2D).

### 4.3 Effects of Carbogen and 100% O_2_ on R_1_ Values and TOLD Signals in Mouse Brains (In Vivo Experiment 1)

**FIGURE 5:**
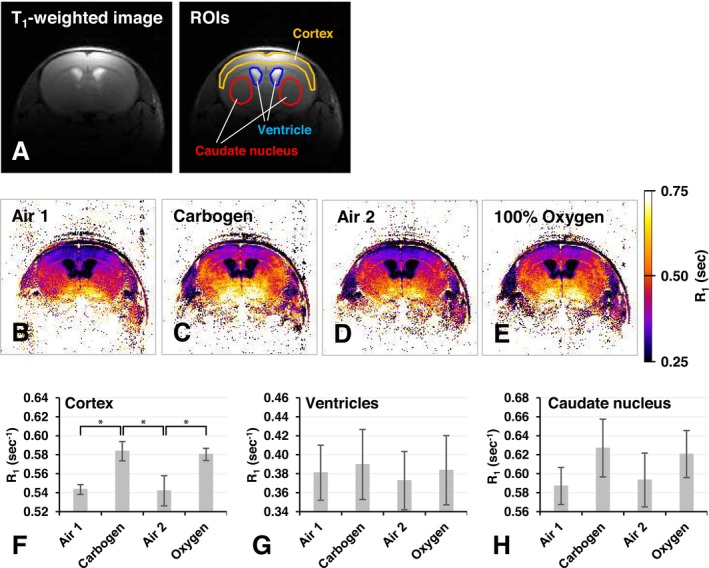
R_1_ maps observed in the brain when the mouse was breathing air, carbogen, or 100% O_2_ and a comparison of R_1_ values in brain regions. (A) T_1_‐weighted image and position of ROI. (B) R_1_ map observed under air‐breathing period 1. (C) R_1_ map observed under the carbogen‐breathing period. (D) R_1_ map observed under air‐breathing period 2. (E) R_1_ map observed under the 100% O_2_‐breathing period. (F) R_1_ values observed in the cortex. (G) R_1_ values observed in the ventricles. (H) R_1_ values observed in the caudate nucleus. Values are indicated as the average ± SD The number of animals used was 3. The asterisk (*) indicates a significant difference (*p* < 0.05) between before and after the gas treatment observed by the *t*‐test with the “tail” was 1 and the “type” was 1.

In vivo experiment 1 was performed to confirm the shortening of T_1_ and the appearance of a positive TOLD signal in the brains of mice breathing a high O_2_ concentration gas. Figure 5A shows a scout image selected from the T_1_‐mapping dataset and several ROIs selected on the brain. The intensity of R_1_ maps of the brains from mice breathing different gases increased and decreased with changes in the O_2_ concentration in the breathing gas (Figure 5B–E). R_1_ values in the cortex were significantly increased by breathing carbogen and 100% O_2_ (Figure 5F). Other regions in the brain, namely, the ventricles (Figure 5G) and caudate nucleus (Figure 5H), showed a similar pattern to that in the cortex. T_1_ and R_1_ values in mouse brain tissue at 7 T under different gas‐breathing conditions are summarized in Table 2.

**TABLE 2:** T_1_ and R_1_ values observed at 7 T in mouse brain regions under several different gas breathing conditions.

Brain region		Air	Carbogen	O_2_
Cortex	T_1_ (s)	1.85 ± 0.03	1.72 ± 0.03*	1.73 ± 0.02*
	R_1_ (s^−1^)	0.54 ± 0.01	0.58 ± 0.01*	0.58 ± 0.01*
Ventricles	T_1_ (s)	2.84 ± 0.19	2.75 ± 0.23	2.79 ± 0.23
	R_1_ (s^−1^)	0.38 ± 0.03	0.39 ± 0.04	0.38 ± 0.04
Caudate nucleus	T_1_ (s)	1.70 ± 0.07	1.60 ± 0.08	1.62 ± 0.07
	R_1_ (s^−1^)	0.59 ± 0.02	0.63 ± 0.03	0.62 ± 0.02

*Note:* Values indicate the average ± SD of three mice. Regarding air conditions, the average of the Air 1 and Air 2 periods were again averaged in three mice. Asterisks (*) indicate a significant difference estimated by the Tukey–Kramer test with α < 0.05, when the value was compared with that under air breathing.

**FIGURE 6:**
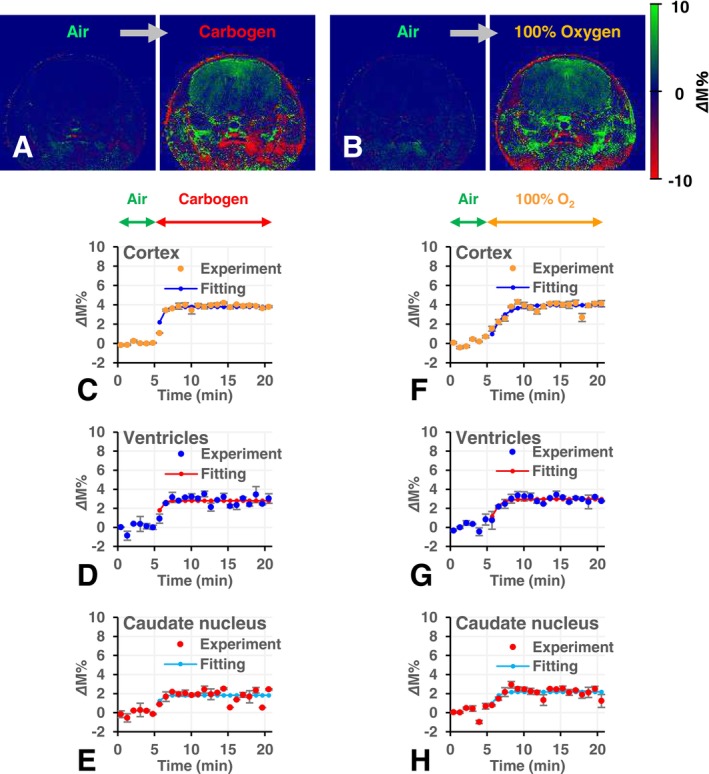
Distribution of the O_2_‐induced T_1_‐weighted signal, that is, the TOLD signal, in the brain of a mouse and the time course of the TOLD signal in ROIs set on brain regions. Positive and negative TOLD signals were induced by switching the carrier gas (A) from air to carbogen and (B) from air to 100% O_2_. The left image is a TOLD signal map observed under air, that is, baseline image, which is the average of the initial 5 frames of 24 frames scanned during 21 min. The right image is the TOLD signal map observed under high concentration O_2_ treatments, and is the average of the last 5 frames of 24 frames. Green and red colors indicate positive and negative TOLD signals. No negative signals were observed in the brain region. Time course of the carbogen‐induced TOLD signal enhancement in the (C) cortex, (D) ventricle, and (E) caudate nucleus regions. Time course of the 100% O_2_‐induced TOLD signal enhancement in the (F) cortex, (G) ventricle, and (H) caudate nucleus regions. Marks with error bars indicate the average ± SD observed in each ROI at each time point. Small marks connected with a line are a simulation of the exponential profile (Equation 5) fit on the experimental values. The signal‐increasing rate, *k*_ox_, and maximum signal level, S_max_, were obtained from the best‐fitting simulation.

Figure 6A,B shows TOLD imaging results. Positive and negative variations in the TOLD signal were observed in the mouse head when the carrier gas for anesthesia was switched from air to carbogen or 100% O_2_. Negative TOLD signals were mainly detected in the lower jaw, which includes muscles and the submandibular gland; however, only a positive TOLD signal was noted in the brain.

Figure 6C–H shows the time courses of TOLD signals in several brain regions. The TOLD signal rapidly increased when the gas was switched from air to carbogen (Figure 6C–E), but more slowly increased when the gas was switched from air to 100% O_2_ (Figure 6F–H). The exponential function (Equation 5) was fit on the increasing phase of the TOLD signal, and the parameters T_ox_ and S_max_ were obtained in the cortex, ventricles, and caudate nucleus under carbogen and 100% O_2_ breathing (Table 3). *k*_ox_ in the cortex was significantly faster under carbogen breathing than under 100% O_2_ breathing. No significant differences were observed in *k*_ox_ in other regions.

**TABLE 3:** Comparison of oxygenation‐related parameters observed in the carbogen‐ or 100% O_2_‐induced TOLD experiment.

Gas tasks	Parameters	Cortex	Ventricles	Caudate nucleus
Carbogen	T_ox_ (min)	0.60 ± 0.16	0.97 ± 0.79	0.66 ± 0.38
	*k*_ox_ (min^−1^)	1.74 ± 0.39*	1.51 ± 0.92	1.83 ± 0.85
	S_max_	5.08 ± 1.14	4.05 ± 1.07	3.08 ± 1.09
100% O_2_	T_ox_ (min)	2.23 ± 1.27	1.70 ± 1.69	1.39 ± 1.48
	*k*_ox_ (min^−1^)	0.54 ± 0.23*	1.05 ± 0.74	1.49 ± 1.23
	S_max_	5.68 ± 1.47	4.07 ± 0.98	3.47 ± 1.12

*Note:* Values indicate the average ± SD of thre mice. Asterisks (*) indicate a significant difference between carbogen and 100% O_2_ estimated by the *t*‐test with *p* < 0.05 with the “tail” was 2 and the “type” was 1 (for paired samples).

### 4.4 Effects of O_2_ on R_1_ and R_2_ Values in Samples

**FIGURE 7:**
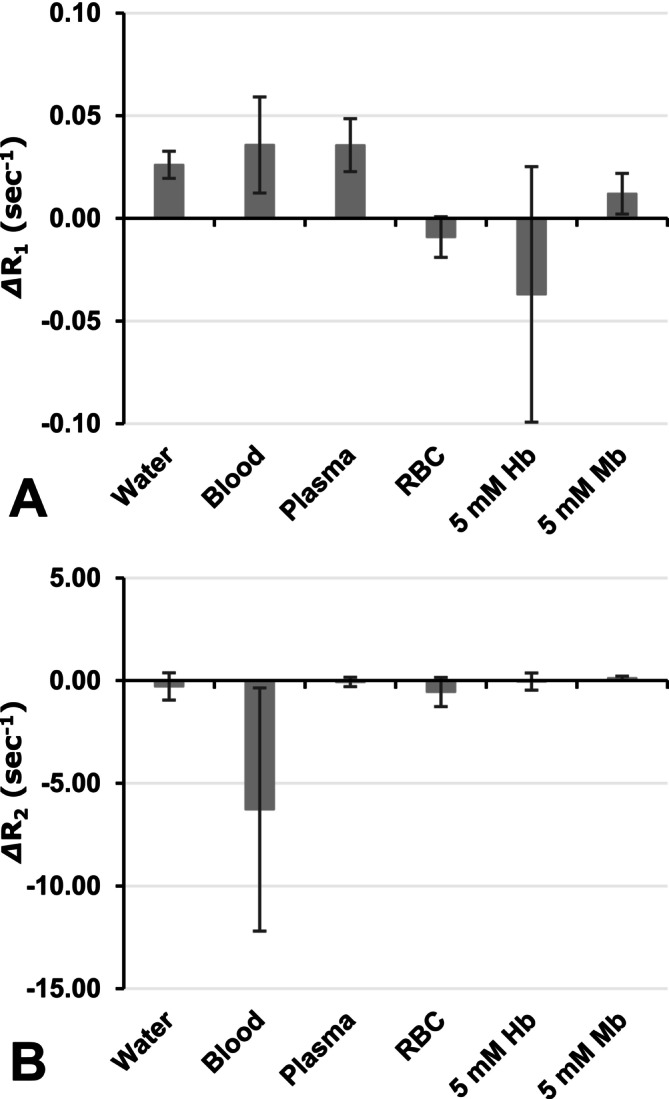
Differences in R_1_ and R_2_ values of aqueous samples between N_2_ and air conditions. Comparison of (A) *Δ*R_1_ and (B) *Δ*R_2_ values obtained in milli‐Q water, whole blood, plasma, the RBC fraction, 5 mM hemoglobin solution, and 5 mM myoglobin solution. The R_1_ and R_2_ values observed under the N_2_ condition were subtracted from the values observed under the air condition. *Δ*R_1_ and *Δ*R_2_ values were obtained for each sample and then averaged. Values indicate the average ± SD of four experiments, while values for water were calculated from six experiments.

R_1_ and R_2_ values in blood samples and solution samples were measured under N_2_ gas flow and also under air flow. A difference in the R_1_ value between N_2_ and air conditions, *Δ*R_1_ = R_1A_ − R_1N_, was obtained, where R_1A_ and R_1N_ are R_1_ values observed under air and under N_2_ flow, respectively. Similarly, *Δ*R_2_ values were obtained. Figure 7A,B shows a comparison of *Δ*R_1_ and *Δ*R_2_ obtained in aqueous samples. Positive and negative *Δ*R_1_ and *Δ*R_2_ values indicate the shortening and extension of T_1_ and T_2_ values, respectively. R_1_ values in milli‐Q water, blood, plasma, and 5 mM Mb solution were increased, that is, T_1_ were shortened, by a small elevation in the concentration of O_2_ by switching gas flow in the sample tube from N_2_ to air. However, R_1_ values in the RBC fraction and 5 mM Hb solution decreased, that is, T_1_ were extended, by increasing the concentration of O_2_. T_2_ values were almost stable with small O_2_ increases, except for T_2_ in blood, which was extended. T_1_, R_1_, T_2_, and R_2_ values are summarized in Table S1.

### 4.5 Effects of Heme Iron on O_2_‐Induced Changes in the Pseudo‐TOLD Signal in Samples

**FIGURE 8:**
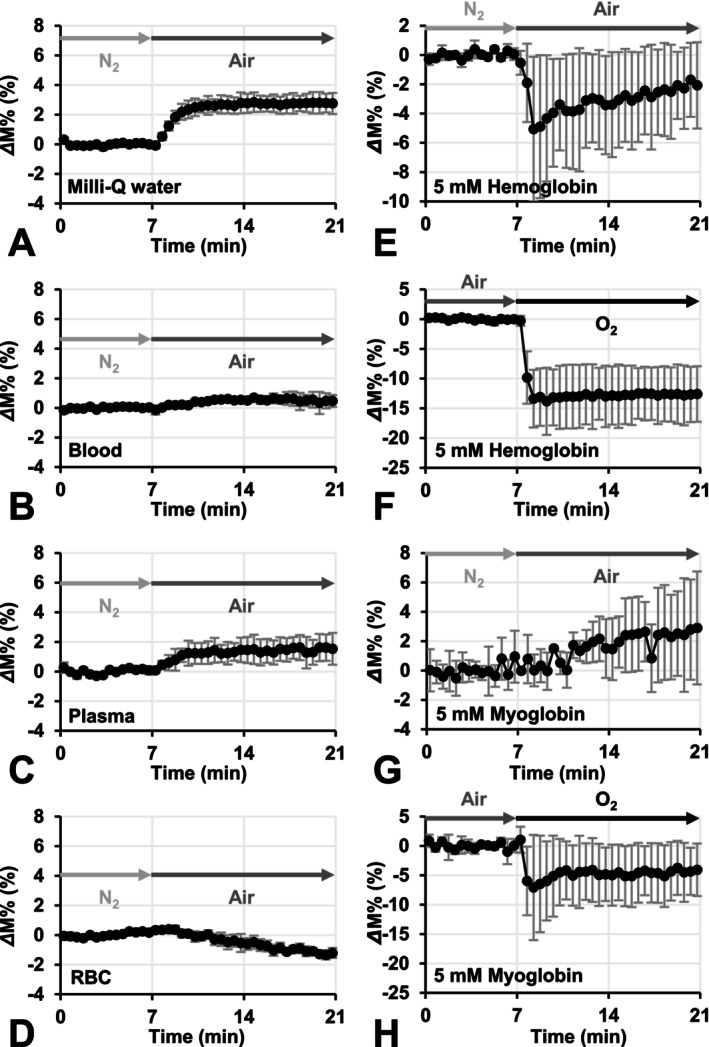
The pseudo‐TOLD signal observed in aqueous samples. The time course of T_1_‐weighted signal enhancement/diminishment (*Δ*M%) was observed in (A) milli‐Q water, (B) mouse whole blood, (C) blood plasma, and (D) the RBC fraction when gas flow in the sample tube was switched from N_2_ to air. The time course of the TOLD signal was also observed in (E, F) 5 mM hemoglobin solution and (G, H) 5 mM myoglobin solution. Experiments in (F) and (H) were performed with air for the initial 7 min followed by a switch to 100% O_2_ gas. Marks with error bars indicate the average ± SD observed at each time point.

Figure 8 shows pseudo‐TOLD signal profiles observed in several samples. When the gas flowing inside the sample tube was switched from N_2_ to air, a positive pseudo‐TOLD signal was observed in milli‐Q water (Figure 8A). In blood, no signal shift or a very slight positive shift was observed (Figure 8B). Plasma also showed a clear positive pseudo‐TOLD signal (Figure 8C). However, a negative pseudo‐TOLD signal was observed in the RBC fraction (Figure 8D). Figure 8E–H shows pseudo‐TOLD signal profiles observed in aqueous solutions of 5 mM Hb and 5 mM Mb. In the 5 mM Hb solution, a negative pseudo‐TOLD signal was observed when gas flow was switched from N_2_ to air (Figure 8E). A larger negative pseudo‐TOLD signal was noted in the 5 mM Hb solution when gas flow was switched from air to O_2_ (Figure 8F). In the 5 mM Mb solution, a positive pseudo‐TOLD signal was observed when gas flow was switched from N_2_ to air (Figure 8G); however, a clear negative pseudo‐TOLD signal was detected in 5 mM Mb (Figure 8H).

### 4.6 In Vivo Experiment 2

**FIGURE 9:**
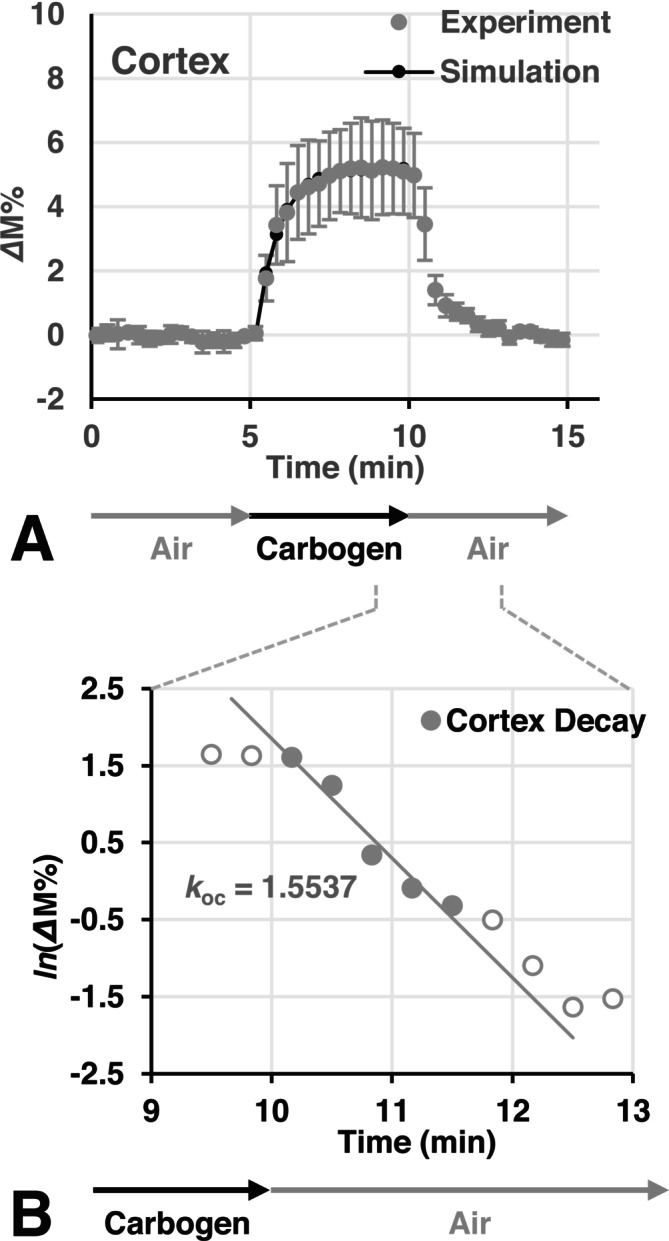
An example of the TOLD response in the brain cortex induced by the carbogen treatment. (A) A TOLD signal response in the brain cortex induced by the carbogen treatment. Equation (5) was fit on the increasing phase of the TOLD signal, and the oxygenation rate, *k*_ox_ and oxygenation level, S_max_ were obtained. Marks and error bars indicate the average ± SD in a ROI. (B) A semi‐logarithmic plot of the decreasing phase of the TOLD signal, and the O_2_ consumption rate *k*_oc_ was obtained from its slope. The slope was estimated using five data points indicated by solid circles.

In vivo experiment 2 attempted to estimate the oxygenation rate, *k*_ox_, oxygenation level, S_max_, and O_2_ consumption rate, *k*_oc_ in the mouse brain cortex. Figure 9A shows an example of the TOLD response observed in the cortex by carbogen breathing. The TOLD signal increased when the breathing gas was switched from air to carbogen and decreased when the gas was switched back to air. *k*_ox_ and S_max_ were estimated by fitting Equation (5) on the increasing phase of the TOLD signal. Figure 9B shows a semi‐logarithmic plot of the decreasing phase of the TOLD signal, and *k*_oc_ was estimated from the slope. *k*_ox_, S_max_, and *k*_oc_ estimated for healthy 8‐week‐old female C3H/HeSlc mice were 1.69 ± 0.44 min^−1^ (*n* = 8), 5.18 ± 1.04% (*n* = 8), and 1.55 ± 0.33 min^−1^ (*n* = 9), respectively.

## 5 Discussion

With the assumption that normal tissue pO_2_ under physiological conditions is 40 mmHg, the R_1_ values of water and corn oil at 40 mmHg pO_2_ were estimated to be 0.220 and 1.546 s^−1^ from the plots shown in Figures 1B and 2B, respectively. Based on the combination ratio of R_1_ values at 40 mmHg in water and corn oil, rough estimations of the aqueous/lipidic ratio of T_1_ components in brain tissue were calculated as 0.76/0.24 in the cortex, 0.88/0.12 in the ventricles, and 0.72/0.28 in the caudate nucleus.

The difference R_1_ values, *Δ*R_1_, in the cortex between air and carbogen breathing was 0.041 s^−1^, while that between air and 100% O_2_ breathing was 0.038 s^−1^. Shifts in pO_2_, *Δ*O_2_, in the cortex induced by carbogen and 100% O_2_ were estimated as 0.28 and 0.26 mmol/L, respectively, when the r_1_ values observed in water and corn oil were used with the ratio of aqueous and lipidic components. Estimated pO_2_ changes in brain regions are summarized in Table 4.

**TABLE 4:** Comparison of *Δ*R1 values in several brain regions in experimental animals breathing carbogen or 100% oxygen.

Breathing gas		Cortex	Ventricles	Caudate nucleus
Carbogen	*Δ*R_1_ (s^−1^)	0.041 ± 0.009	0.013 ± 0.007	0.037 ± 0.008
	mM equivalent	0.28	0.098	0.24
	mmHg equivalent	194	68	168
Oxygen	*Δ*R_1_ (s^−1^)	0.038 ± 0.009	0.007 ± 0.007	0.031 ± 0.005
	mM equivalent	0.26	0.052	0.20
	mmHg equivalent	178	36	139

*Note: Δ*R_1_ values indicate the average ± SD The number of animals used was 3. Other values are calculated with the average values of *Δ*R_1_. To calculate pO_2_ changes, r_1_ values observed in water, 1.71 × 10^−4^ mmHg^−1^ s^−1^ and 0.118 mM^−1^ s^−1^, and in corn oil, 8.20 × 10^−4^ mmHg^−1^ s^−1^ and 0.569 mM^−1^ s^−1^, were used with the ratio of aqueous and lipidic components in the corresponding brain region.

As shown in Equation (1), a tissue T_1_ value estimated by T_1_ mapping is a compression of several T_1_ components. When tissue pO_2_ increases, O_2_ itself may shorten tissue T_1_ due to the stable unpaired electron on it. At the same time, transformation from deoxy‐Hb to oxy‐Hb may extend T_1_ in blood due to the loss of paramagnetism. Similarly, in muscle, transformation from deoxy‐Mb to oxy‐Mb exerts similar effects. Samples with negative *Δ*R_1_ changes in Figure 7, that is, the RBC fraction and 5 mM Hb solution, showed a negative pseudo‐TOLD signal in Figure 8. The 5 mM Mb solution did not show a negative TOLD signal by small O_2_ increases, that is, a shift from N_2_ to air (Figure 8G); however, larger O_2_ increases, that is, a shift from air to O_2_, induced a negative TOLD signal in the 5 mM Mb solution (Figure 8H). Mb, which has only one heme, showed smaller effects than Hb, which has four hemes, at the same concentration. Similarly, whole blood showed smaller effects than the RBC fraction.

The brain parenchyma is tightly separated from blood components by the blood–brain barrier. The content of heme iron, a component related to T_1_ extensions under oxygenation, in the physiological state is almost zero in the brain parenchyma. Heme iron is exclusively presented to circulating blood, and the BOLD effect may be detected in circulating blood containing a large amount of O_2_ bound to Hb. On the other hand, the TOLD effect may be observed by transmitted free O_2_ molecules in the brain parenchyma.

*Δ*R_1_ values in brain tissue increased and decreased in synchrony with oxygenation tasks (Figure 5F–H). *Δ*R_1_ values in brain tissue were similar under carbogen and 100% O_2_ breathing. The TOLD signal in brain tissue also synchronized with oxygenation tasks (Figures 6 and 9A). The oxygenation phase and O_2_ consumption phase in the brain may be assessed (Figure 9). Furthermore, S_max_ values in brain tissue showed similar increases under carbogen and 100% O_2_ breathing (Figure 6C–H and Table 3). These results suggest that a positive TOLD signal in the brain reflects increasing pO_2_ in brain tissue.

In in vivo experiment 1, the levels of enhancement in the R_1_ and T_1_‐weighted signals, that is, *Δ*R_1_ and S_max_, induced by carbogen and 100% O_2_ were measured. Observations of differences in oxygenation rates, *k*_ox_, between carbogen breathing and 100% O_2_ breathing provided the parameters for in vivo experiment 2, which involved a kinetic analysis of tissue O_2_ levels. Breathing carbogen was selected for the oxygenation task in in vivo experiment 2 because breathing 100% O_2_ delayed the distribution of O_2_.

O_2_ molecules easily permeate the brain–blood barrier and are quickly distributed throughout brain tissue. However, breathing carbogen results in the faster distribution of O_2_ throughout brain tissue than breathing 100% O_2_. The *k*_ox_ value, that is, the enhancement rate of the TOLD signal, was faster under breathing carbogen than under breathing 100% O_2_, with a significant increase being observed in the cortex (Table 3). Breathing 100% O_2_ has been shown to induce vascular constriction, while breathing 5% carbon dioxide in carbogen gas caused vasodilatation, which increased blood flow [39]. Therefore, the O_2_ distribution rate may be reduced under breathing 100% O_2_ and increased under breathing carbogen.

The decay rate of the TOLD signal, observed when the breathing gas was switched from carbogen to air, reflects O_2_ consumption by brain tissue. Excess O_2_ in the brain was quickly consumed (Figure 9B). The O_2_ consumption rate observed in the brain in the present study was 1.55 ± 0.33 min^−1^, which was markedly faster than that observed in muscle, even when the O_2_ supply to the brain remained. pO_2_ in the mouse leg muscle rapidly decreased to less than 2 mmHg within 5 min when the O_2_ supply was disrupted [40]. O_2_ consumption rates in muscle were recalculated based on the data shown in fig. 6 of [40] as 0.46 or 0.83 min^−1^.

When the gas that the experimental animal was breathing was switched from air to carbogen during a repeating T_1_‐weighted scan, it was possible to observe the time course of increases in the TOLD signal and quantify the tissue oxygenation rate, *k*_ox_. Similarly, when the gas was switched from carbogen to air, it was possible to observe the time course of decreases in the TOLD signal and quantify the O_2_ consumption rate, *k*_oc_. Although this method requires further improvements for the more accurate estimation of O_2_ consumption rates, the parameters observed in the TOLD experiment, namely, *k*_oc_, *k*_ox_, and S_max_, are useful for digitizing biological effects in the tissues of living experimental animals. This is the first study to perform a kinetic analysis of O_2_ levels in normal healthy mouse brains based on variations in the T_1_‐weighted signal, that is, the TOLD signal, induced by an oxygen challenge.

## 6 Conclusion

In mice breathing carbogen or 100% O_2_, the R_1_ value and TOLD signal increased in brain tissue. A negative TOLD signal was not observed in the brain. Similar changes in R_1_ values and the TOLD signal were noted in mice breathing carbogen and 100% O_2_. A positive TOLD signal in the brain reflects the rapid uptake and distribution of O_2_. A method for a time course analysis of the TOLD signal was herein proposed. The results obtained indicate that the negative shift in the TOLD MRI signal in mouse muscle tissues under O_2_ breathing was due to a shift in paramagnetic deoxy‐Hb to diamagnetic oxy‐Hb.

In the present study, estimations of the O_2_ consumption rate in the mouse brain were performed by analyzing the time course of the TOLD signal. The TOLD signal in the brain was increased by carbogen breathing and decreased to the baseline level when the breathing gas was switched back to air. An increase in the rate of the TOLD signal, that is, the oxygenation rate *k*_ox_ and the maximum signal intensity, S_max_, may be obtained by fitting an exponential curve to the increasing phase of the TOLD signal. The O_2_ consumption rate, *k*_oc_, was estimated as the slope of a semilogarithmic plot of the decreasing phase of the TOLD signal. A method to quantify oxygenation and O_2_ consumption rates based on TOLD imaging is proposed as a diagnostic tool for estimating the distribution and consumption of O_2_ in brain tissue.

## Conflicts of Interest

The authors declare no conflicts of interest.

## Supporting information


**Figure S1.** Schematic drawing of sample tube settings of X‐band EPR measurements under hypoxic conditions. A hole (1.1 mm in diameter) was made on the bottom of a quartz sample holder (inner diameter of 3.85 mm at the upper entrance and the lower half is slimmer than the upper half). Plastic tubing was connected to the bottom of the quartz sample holder, and air, N_2_, or O_2_ gas was flowed at a rate of 1 L/min. The quartz sample holder was fixed in the X‐band EPR cavity. The position of LiPc crystals was adjusted to the center of the sensitive region in the cavity.
**Figure S2.** Sample settings of an experiment for estimating the T_1_ relaxivity (r_1_) of O_2_ dissolved in water or corn oil. A 24‐cm piece of gas‐permeable PTFE tubing enclosing an ~80‐μL aliquot of water or corn oil was fixed inside quartz tubing. N_2_ (0% O_2_), air (21% O_2_), carbogen (95% O_2_), or 100% O_2_ gas was flowed (0.4 mL/min) inside quartz tubing (i.d. = 3.6 mm). Temperature was measured and controlled by warmed air flow outside quartz tubing.
**Figure S3.** Flow diagrams of experiments 1 (left panel) and 2 (right panel). Experiment 1 took approximately 1 h 40 min, while experiment 2 took approximately 15 min.
**Figure S4.** Relationship between the EPR linewidth of LiPc and O_2_ concentrations in water. Marks and error bars indicate the average ± SD of 3 experiments. Small error bars are obscured by the marks.

**Table S1.** Comparison of T_1_, R_1_, T_2_, and R_2_ values observed at 7 T in mouse whole blood, plasma, and the RBC fraction under N_2_ or air saturation.

## Data Availability

The data that support the findings of this study are available from the corresponding author upon reasonable request.
