# Supplementary material for: Quantification of Oxygenation and Oxygen Consumption Rates in the Mouse Brain Based on Tissue Oxygen Level‐Dependent (TOLD) MRI
Source: NMR Biomed. 2025 Jul 22;38(9):e70104. doi: 10.1002/nbm.70104 (PMC12281473; doi:10.1002/nbm.70104)
Supplement: Supplementary file 1 — Figure S1. Schematic drawing of sample tube settings of X‐band EPR measurements under hypoxic conditions. A hole (1.1 mm in diameter) was made on the bottom of a quartz sample holder (inner diameter of 3.85 mm at the upper entrance and the lower half is slimmer than the upper half). Plastic tubing was connected to the bottom of the quartz sample holder, and air, N2, or O2 gas was flowed at a rate of 1 L/min. The quartz sample holder was fixed in the X‐band EPR cavity. The position of LiPc crystals was adjusted to the center of the sensitive region in the cavity. Figure S2. Sample settings of an experiment for estimating the T1 relaxivity (r1) of O2 dissolved in water or corn oil. A 24‐cm piece of gas‐permeable PTFE tubing enclosing an ~80‐μL aliquot of water or corn oil was fixed inside quartz tubing. N2 (0% O2), air (21% O2), carbogen (95% O2), or 100% O2 gas was flowed (0.4 mL/min) inside quartz tubing (i.d. = 3.6 mm). Temperature was measured and controlled by warmed air flow outside quartz tubing. Figure S3. Flow diagrams of experiments 1 (left panel) and 2 (right panel). Experiment 1 took approximately 1 h 40 min, while experiment 2 took approximately 15 min. Figure S4. Relationship between the EPR linewidth of LiPc and O2 concentrations in water. Marks and error bars indicate the average ± SD of 3 experiments. Small error bars are obscured by the marks. [file NBM-38-e70104-s002.pdf]

Supplementary Figure 1

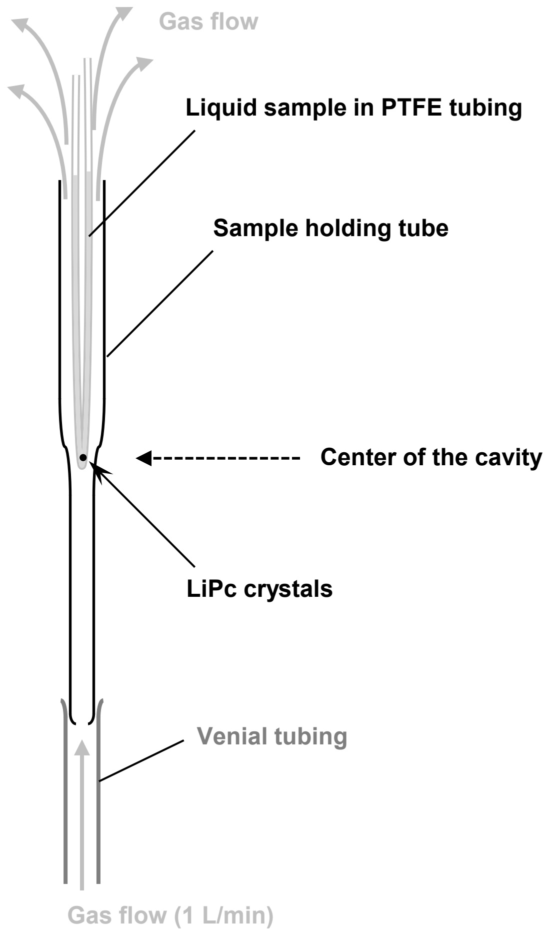

Suppl. Fig. 1. Schematic drawing of sample tube settings of X-band EPR measurements under hypoxic conditions. A hole (1.1 mm in diameter) was made on the bottom of a quartz sample holder (inner diameter of 3.85 mm at the upper entrance and the lower half is slimmer than the upper half). Plastic tubing was connected to the bottom of the quartz sample holder, and air, N_2, or O_2 gas was flowed at a rate of 1 L/min. The quartz sample holder was fixed in the X-band EPR cavity. The position of LiPc crystals was adjusted to the center of the sensitive region in the cavity.

Supplementary Figure 2

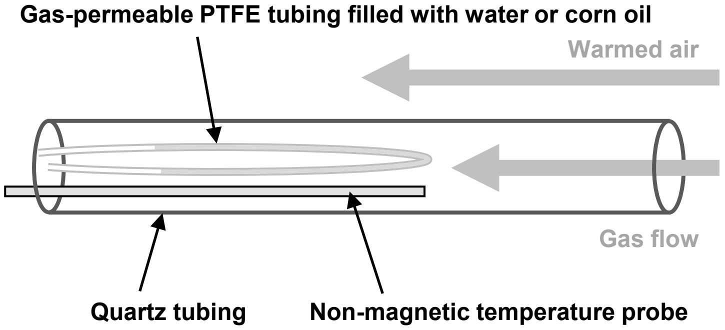

Suppl. Fig. 2. Sample settings of an experiment for estimating the T_1 relaxivity (r_1) of O_2 dissolved in water or corn oil. A 24-cm piece of gas-permeable PTFE tubing enclosing an ~80-µL aliquot of water or corn oil was fixed inside quartz tubing. N_2 (0% O_2), air (21% O_2), carbogen (95% O_2), or 100% O_2 gas was flowed (0.4 mL/min) inside quartz tubing (i.d. = 3.6 mm). Temperature was measured and controlled by warmed air flow outside quartz tubing.

Supplementary Figure 3

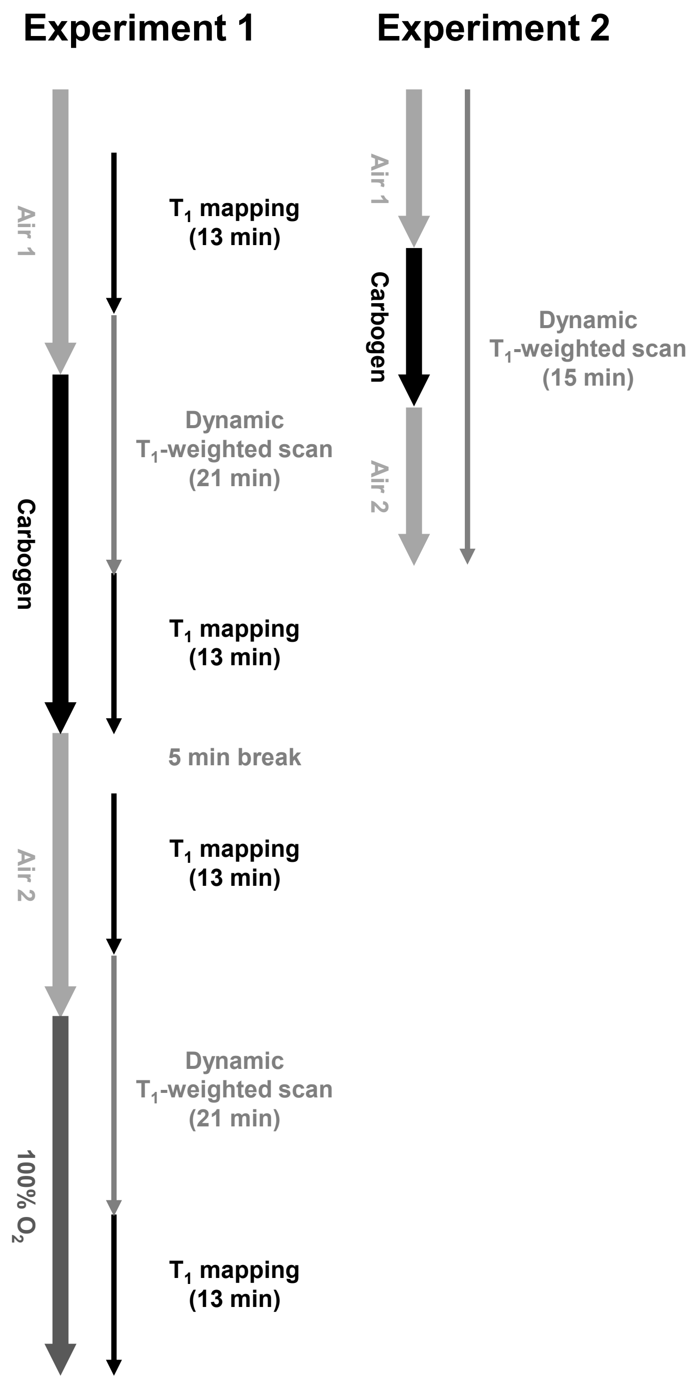

Suppl. Fig. 3. Flow diagrams of experiments 1 (left panel) and 2 (right panel). Experiment 1 took approximately 1 hr 40 min, while experiment 2 took approximately 15 min.

Supplementary Figure 4

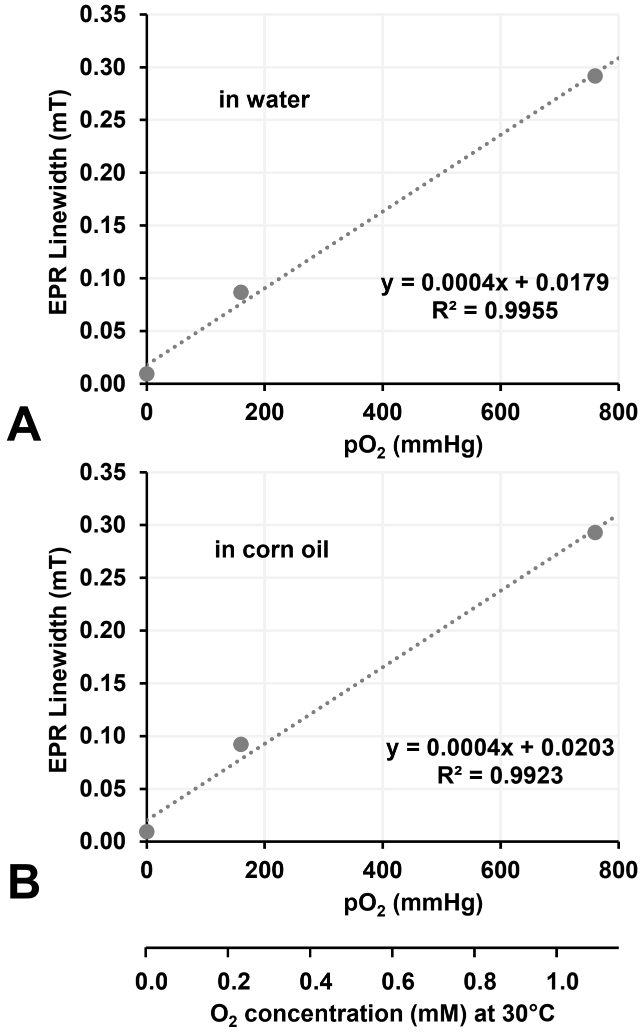

Suppl. Fig. 4. Relationship between the EPR linewidth of LiPc and O_2 concentrations in water. Marks and error bars indicate the average $\pm$ SD of 3 experiments. Small error bars are obscured by the marks.
