# Supplementary material for: Quantification of Oxygenation and Oxygen Consumption Rates in the Mouse Brain Based on Tissue Oxygen Level‐Dependent (TOLD) MRI
Source: NMR Biomed. 2025 Jul 22;38(9):e70104. doi: 10.1002/nbm.70104 (PMC12281473; doi:10.1002/nbm.70104)
Supplement: Supplementary file 2 — Table S1. Comparison of T1, R1, T2, and R2 values observed at 7 T in mouse whole blood, plasma, and the RBC fraction under N2 or air saturation. [file NBM-38-e70104-s001.pdf]

Suppl. Table 1. Comparison of T_1, R_1, T_2, and R_2 values observed at 7 T in mouse whole blood, plasma, and the RBC fraction under N_2 or air saturation.

Sample	Gas	T_1 (s)	R_1 (s^{-1})	T_2 (s)	R_2 (s^{-1})
Water	N_2	5.167 ± 0.438	0.217 ± 0.030	0.188 ± 0.013	5.776 ± 0.837
	Air	4.642 ± 0.302**	0.235 ± 0.026	0.189 ± 0.018	5.496 ± 0.486
Blood	N_2	2.764 ± 0.262	0.378 ± 0.040	0.053 ± 0.016	21.546 ± 8.157
	Air	1.345 ± 1.292*	0.220 ± 0.222	0.070 ± 0.014*	15.271 ± 3.318
Plasma	N_2	3.645 ± 0.227	0.285 ± 0.029	0.129 ± 0.013	7.870 ± 0.668
	Air	3.390 ± 0.030	0.303 ± 0.019	0.131 ± 0.010	7.803 ± 0.473
RBC	N_2	1.684 ± 0.042	0.596 ± 0.016	0.0365 ± 0.0094	28.800 ± 6.550
	Air	1.711 ± 0.048	0.587 ± 0.018	0.0374 ± 0.0092*	28.246 ± 6.077
5 mM Hb	N_2	0.187 ± 0.009	5.389 ± 0.277	0.0360 ± 0.0028	28.017 ± 2.016
	Air	0.188 ± 0.011	5.352 ± 0.339	0.0360 ± 0.0014	27.970 ± 1.597
5 mM Mb	N_2	0.474 ± 0.008	2.115 ± 0.035	0.0815 ± 0.0007	12.295 ± 0.039
	Air	0.471 ± 0.048	2.127 ± 0.025	0.0810 ± 0.0014	12.384 ± 0.209

Values indicate the average ± SD of 4 experiments, while values for water were calculated from 6 experiments. Asterisks (*) indicate a significant difference between N_2 and air estimated by the *t*-test with $p < 0.05$, when the values were compared as pairs. Water is milli-Q water.
